# Potential Predictive Value of Platelet Parameters in Preeclampsia

**DOI:** 10.1089/whr.2023.0162

**Published:** 2024-05-21

**Authors:** Peng Li, Hui Chen, Xiaoyun Zhang, Jishui Wang, Yuting Li, Yanping Wang, Fengge Wang, Dongmei Man

**Affiliations:** ^1^ College of Clinical Medicine, Jining Medical University, Jining, Republic of China.; ^2^ Department of Obstetrics, Affiliated Hospital of Jining Medical University, Jining Medical University, Jining, Republic of China.

**Keywords:** preeclampsia, platelet parameters, pregnancy, platelet

## Abstract

**Objective:**

Preeclampsia is a serious pregnancy complication that jeopardizes the health of both the mother and the fetus. Platelet parameters are closely linked to the severity of preeclampsia. This study aimed to assess the diagnostic potential of platelet parameters in the early second trimester for the detection of preeclampsia.

**Methods:**

A total of 840 participants from the Affiliated Hospital of Jining Medical College were included in the study, consisting of 327 healthy pregnant women, 209 with mild preeclampsia, and 304 with severe preeclampsia. General clinical data and platelet parameters for these three groups of pregnant women were collected, and differences among them were compared. In addition, univariate analysis and logistic regression were used to identify preeclampsia risk factors, and receiver operating characteristic curve analysis was conducted to assess the predictive value of platelet parameters.

**Results:**

Platelet count was not found to significantly differ between the healthy and preeclampsia groups. However, mean platelet volume, platelet distribution width (PDW), and platelet–large cell ratios (P-LCR) were observed to be significantly higher in the preeclampsia group than the healthy group. After adjusting for confounding factors (such as age, gestational week at blood sampling, systolic and diastolic blood pressure, and body mass index during the second trimester), it was determined that PDW and P-LCR could be considered effective predictors of preeclampsia.

**Conclusion:**

In clinical practice, P-LCR and PDW hold potential predictive value for preeclampsia.

## Introduction

Preeclampsia is an inexplicable condition characterized by multiple factors occurring during pregnancy. It can affect approximately 3%–5% of pregnancies, resulting in the annual loss of 76,000 women and 500,000 infants worldwide.^1^ Preeclampsia’s defining features include high blood pressure and endothelial dysfunction, which can lead to significant damage to various vital organs, including the liver, kidneys, brain, placenta, and cardiovascular system.^2^ Developing countries, in particular, face elevated maternal and perinatal morbidity and mortality due to limited medical resources and reduced access to adequate obstetric care.^3^ Despite advancements in medical care, the impact of preeclampsia remains unabated.^3^ Research has revealed that pregnant women with preeclampsia face a heightened risk of developing cardiovascular and cerebrovascular disorders in the future.^4,5^ Hence, the urgent need for an early predictor of preeclampsia to reduce maternal and fetal mortality.^6^

Preeclampsia is primarily associated with the placenta, and the only curative intervention is placental delivery.^1^ To date, the exact pathogenesis of preeclampsia remains unclear. However, substantial evidence supports the “two-stage” theory of preeclampsia.^7,8^ The first stage, known as the preclinical stage, is marked by insufficient invasion of gestational trophoblast cells into uterine tissues, resulting in impaired remodeling of maternal spiral arterioles. This, in turn, leads to placental hypoperfusion, subsequent ischemia, hypoxia, and increased release of placental factors such as chemokines, proinflammatory cytokines, and antiangiogenic factors.^7^ During the second stage, these placental factors enter the maternal circulation, triggering a systemic inflammatory response and causing vascular endothelial damage, which manifests in various clinical features of preeclampsia.^7^ When endothelial injury occurs, platelets adhere to the damaged endothelial cells, become activated, and release contents from alpha and dense granules, further promoting platelet aggregation.^9^ Moreover, damaged vascular endothelium can release tissue factor, initiating the coagulation process.^9^ The excessive activation of the hemostatic system and heightened platelet aggregation result in multiorgan hypoperfusion and multisystem dysfunction in preeclampsia.^10^ Abnormal coagulation in preeclampsia leads to elevated platelet consumption, compelling the bone marrow to produce a surplus of younger and larger platelets, consequently affecting platelet parameters in maternal circulation, including platelet count (PC), mean platelet volume (MPV), platelet distribution width (PDW), and platelet–large cell ratios (P-LCR).^11^ In clinical practice, platelet parameters serve as markers of platelet activation and play a pivotal role in predicting and diagnosing various medical conditions, including cardiovascular and hematological disorders.^12^ These platelet parameters are part of a routine complete blood count and are both convenient and cost-effective to obtain.^13^ However, studies investigating whether platelet parameters can predict preeclampsia have yielded inconsistent conclusions, and the existing research is limited by small sample sizes and systematic errors. This study addresses these limitations by utilizing a large sample size to explore the predictive value of platelet parameters for preeclampsia through a comprehensive comparison of platelet parameters between preeclamptic and normotensive pregnant women.

## Methods

### Study subjects

**FIG. 1.**
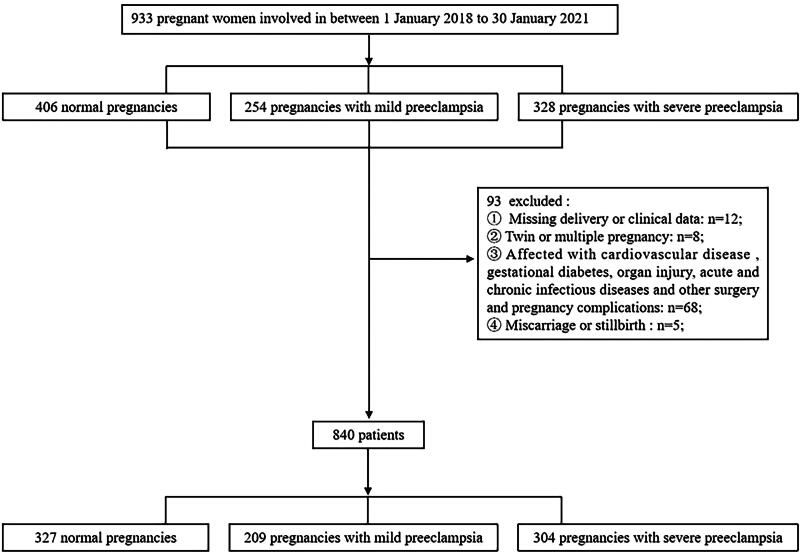
Flowchart of the study population.

This study involved a total of 933 pregnant women, who were enrolled between January 1, 2018, and January 30, 2021 (Fig. 1). We state that these 933 pregnant women do not represent the total number of pregnant women who attended the hospital between January 1, 2018, and January 30, 2021.

Healthy pregnancies were defined as those in which normal blood pressure and the absence of complications such as gestational diabetes, hypothyroidism, or fetal cardiac abnormalities during the perinatal period were observed in age-matched pregnant women. Preeclampsia was characterized by the new onset of hypertension after 20 weeks of pregnancy, with systolic blood pressure ≥140 mmHg and/or diastolic blood pressure ≥90 mmHg. It was accompanied by urinary protein quantification ≥0.3 g per 24 hours, urinary protein–creatinine ratio ≥0.3, or random urinary protein ≥ (+). In addition, the diagnosis of preeclampsia extended to women with gestational hypertension without proteinuria but with the presence of abnormal changes in vital organs, such as the heart, lungs, liver, kidneys, or complications in the hematological, digestive, nervous, and placenta–fetal systems.

The progression to worsening preeclampsia was characterized by continued increases in blood pressure or urinary protein levels, the development of organ dysfunction in pregnant women, and the occurrence of placental-fetal complications.

Severe preeclampsia could be diagnosed when any of the following adverse conditions were met: systolic blood pressure ≥160 mmHg and/or diastolic blood pressure ≥110 mmHg, urinary protein ≥2 g per 24 hours, or random urinary protein ≥ (+++). Furthermore, indications of severe preeclampsia included persistent symptoms such as headache, visual impairment, dizziness, blurred vision, right epigastric pain, liver dysfunction, renal dysfunction, hypoproteinemia, abnormal blood system, heart failure, pulmonary edema, fetal intrauterine growth restriction, or oligohydramnios.

Eclampsia is characterized by tetanic convulsions that occur as a result of preeclampsia and cannot be attributed to other causes. It can manifest during the antepartum, intrapartum, or postpartum period, even in the absence of clinical signs of preeclampsia.

Exclusion criteria of the study were as follows:

1. Pregnant women with a history of hypertension, cardiovascular disease, diabetes, kidney or liver injury, thromboembolism, known thrombophilic disease, hyperthyroidism, or the use of medications affecting PC and platelet function were excluded.
2. Patients with twin or multiple pregnancies, hydatidiform molar pregnancies, inadequate clinical data, recent major surgeries or trauma, and pre-pregnancy body mass index (BMI) ≥30 kg/m^2^ were also excluded.
3. Any pregnancy complicated by fetal growth restriction, premature delivery, recurrent miscarriage, intrauterine fetal death, acute pyelonephritis, premature rupture of membranes, or infection was excluded owing to the potential impact on platelet parameters.

Clinically, the diagnosis of preeclampsia occurs after the 20th week of gestation. We aimed to investigate the predictive value of platelet parameters before the onset of preeclampsia and therefore selected platelet parameters obtained around the 16th week of gestation. According to the standard operation process, under aseptic conditions, 4 ml of blood was collected in ethylene diamine tetra acetic acid (EDTA) vials. Platelet parameters of the sample were determined within 2 hours of blood collection using an automated hematology analyzer. We obtained patient information from the Haitai Medical Archives Information System while strictly adhering to patient privacy protection principles, ensuring the confidentiality of pregnant women’s personal information. This study received approval from the Ethics Committee of the Affiliated Hospital of Jining Medical College, and all patients provided informed consent.

### Statistical analysis

Continuous variables were expressed as mean ± standard deviation or median, and categorical variables were presented as frequency or percentage. Group comparisons were conducted using chi square tests, one-way analysis of variance, or the Kruskal–Wallis test. Logistic regression was used to identify factors influencing preeclampsia. In addition, receiver operating characteristic (ROC) curves were generated to assess the diagnostic value of relevant parameters. Data analysis was performed using the statistical software packages R (http://www.R-project.org, The R Foundation) and EmpowerStats (http://www.empowerstats.com, X&Y Solutions, Inc., Boston, MA). A significance level of *p* < 0.05 with a 95% confidence interval was considered statistically significant.

## Results

### Clinical characteristics of study participants

A total of 840 pregnant women were enrolled in this study, comprising 327 in the healthy pregnancy group, 209 in the mild preeclampsia group, and 304 in the severe preeclampsia group. The fundamental clinical characteristics of these three groups are detailed in Table 1.

**Table 1.** Baseline Characteristics of Subjects

Characteristic	Healthy pregnant controls (*n* = 327)	Mild preeclampsia (*n* = 209)	Severe preeclampsia (*n* = 304)	*p*-value
Age (years)	29.74 ± 5.23	30.70 ± 6.04	31.90 ± 6.35	**<0.001**
BMI	22.95 ± 3.66	22.98 ± 7.27	22.90 ± 9.11	**<0.001**
Gestational week at the time of blood sampling (week)	14.81 ± 2.37	15.11 ± 3.49	16.79 ± 3.90	**<0.001**
Delivery pregnancy week (week)	38.71 ± 2.10	36.14 ± 2.90	34.02 ± 3.93	**<0.001**
Systolic blood pressure (mmHg)	120.43 ± 11.44	165.68 ± 14.54	179.73 ± 16.89	**<0.001**
Diastolic blood pressure (mmHg)	73.06 ± 9.26	103.40 ± 8.75	112.30 ± 10.95	**<0.001**
Neonatal weight (kg)	3.34 ± 0.53	2.64 ± 0.89	2.20 ± 0.85	**<0.001**
PC	261.91 ± 53.60	248.77 ± 61.98	257.05 ± 66.25	0.194
MPV	10.04 ± 1.20	10.48 ± 0.93	10.52 ± 1.07	**<0.001**
P-LCR	24.48 ± 4.96	29.74 ± 6.08	30.61 ± 5.35	**<0.001**
PDW	11.10 ± 1.11	12.61 ± 1.91	12.65 ± 1.52	**<0.001**

Results in table: mean + SD / *n* (%).

Kruskal–Wallis rank test for continuous variables in table.

*p* < 0.05 statistically significant. Statistically significant *p*-values are marked as bold text.

BMI, body mass index; MPV, mean platelet volume; PC, platelet count; PDW, platelet distribution width; P-LCR, platelet–large cell ratios.

Significant variations were observed in age, systolic blood pressure, diastolic blood pressure, gestational weeks at delivery, and BMI during the second trimester among the three groups (*p* < 0.001). However, there was no notable difference in PC among the three groups (*p* = 0.194, > 0.05). Compared with the healthy pregnancy group, pregnant women in the preeclampsia groups exhibited higher age, BMI during the second trimester, systolic blood pressure, and diastolic blood pressure, whereas neonatal weight and the gestational week at delivery were lower. Particularly noteworthy was the significant difference in P-LCR, MPV, and PDW among the three groups (*p* < 0.01).

### Platelet parameters among study groups

Table 2 presents the results of univariate analysis of platelet parameters, indicating a positive correlation between MPV, P-LCR, and PDW levels with preeclampsia (β > 1, *p* < 0.05). Table 3 conducts multiple regression analyses of platelet parameters, providing effect values (odds ratio [OR]) and 95% confidence intervals. Evidently, P-LCR and PDW can effectively predict preeclampsia. In the unadjusted model, the risk of developing preeclampsia increased by 23% (OR: 1.23; 95% CI: 1.17–1.30; *p* < 0.05) and 142% (OR: 2.42; 95% CI: 1.97–2.96; *p* < 0.05) for each unit increase in P-LCR and PDW levels, respectively. After adjusting for a range of factors (age, gestational week at the time of blood sampling, systolic blood pressure, diastolic blood pressure, and BMI during the second trimester), the risk of developing preeclampsia increased by 34% (OR: 1.34; 95% CI: 1.08–1.66; *p* < 0.05) and 351% (OR: 4.51; 95% CI: 1.57–12.96; *p* < 0.05) for each unit increase in P-LCR and PDW levels, respectively.

**Table 2.** Univariate Analysis of Different Variables for Preeclampsia

Covariate	β(95% CI)	*p*-Value
Age	1.07 (1.03, 1.12)	**<0.001**
Systolic blood pressure (mmHG)	1.37 (1.23, 1.52)	**<0.001**
Diastolic blood pressure (mmHG)	1.42 (1.29, 1.56)	**<0.001**
PC	1.00 (0.99, 1.00)	0.026
MPV	1.76 (1.29, 2.40)	**<0.001**
P-LCR	1.23 (1.16, 1.29)	**<0.0001**
PDW	2.41 (1.93, 3.03)	**<0.0001**
Delivery pregnancy week (week)	0.65 (0.58, 0.73)	**<0.0001**
Neonatal weight (kg)	0.21 (0.14,0.32)	**<0.0001**
BMI	1.04 (0.99, 1.09)	0.109

*p* < 0.05 is statistically significant. Statistically significant *p*-values are marked as bold text.

CI, confidence interval; NS; not significant.

**Table 3.** Multivariate Regression Equations of Platelet Parameters for Preeclampsia

Variable	Crude model		Adjusted	
	OR (95% CI)	*p*-value	OR (95% CI)	*p*-value
PC	1.00 (0.99, 1.00)	0.1086	0.96 (0.94, 0.99)	0.0039
MPV	1.69 (1.29, 2.21)	0.0001	1.25 (0.93, 1.68)	0.1446
P-LCR	1.23 (1.17, 1.30)	<0.0001	1.34 (1.08, 1.66)	**0.0081**
PDW	2.42 (1.97, 2.96)	<0.0001	4.51 (1.57, 12.96)	**0.0052**

Logistic regression model (binary logistic regression with single-categorical and multicategorical predictors) was used to determine the possible risk factors for preeclampsia. Adjusted: Adjusted for age, gestational week at time of blood sampling, systolic blood pressure, diastolic blood pressure, and BMI during the second trimester.

*p* < 0.05 is statistically significant. Statistically significant *p*-values are marked as bold text.

OR, odds ratio.

### Diagnostic values of platelet parameters for preeclampsia

**FIG. 2.**
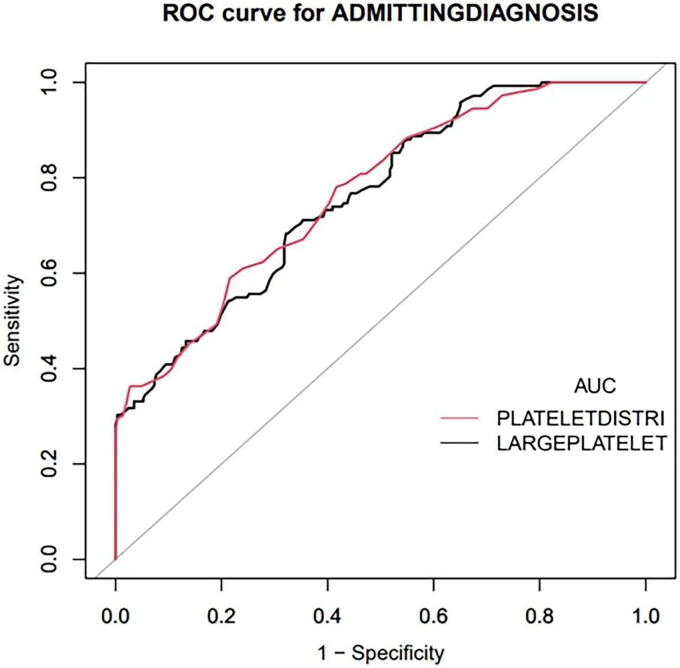
ROC curve of platelet parameters. ROC, receiver operating characteristic.

The ROC curve was utilized to find the optimal threshold values for platelet parameters to predict preeclampsia. As displayed in Figure 2 and Table 4, the analysis confirmed that P-LCR effectively distinguishes preeclamptic pregnant individuals from healthy pregnant ones at a threshold of 27.35%, with a sensitivity of 68.3% and a specificity of 67.8%. At this threshold, the prediction accuracy, positive predictive value (PPV), and negative predictive value (NPV) were 68.0%, 51.3%, and 81.2%, respectively. The analysis also revealed that PDW can differentiate preeclamptic pregnant individuals from healthy pregnant ones at a threshold of 12.05 fl, with a sensitivity of 59.0% and a specificity of 78.5%. At this threshold, the prediction accuracy, PPV, and NPV were 71.9%, 58.1%, and 79.0%, respectively. In addition, the area under the ROC curve for P-LCR and PDW was 0.760 (95% CI: 0.714–0.807) and 0.767 (95% CI: 0.721–0.813), respectively.

**Table 4.** Results of ROC Analysis of Platelet Parameters in Predicting PE

Variable	AUC	95% CI	Cutoff	Sensitivity (%)	Specificity (%)	PPV (%)	NPV (%)	Accuracy
P-LCR	0.760	0.714–0.807	27.350	0.683	0.678	0.513	0.812	0.680
PDW	0.767	0.721–0.813	12.050	0.589	0.785	0.581	0.790	0.719

AUC, area under the curve; P-LCR, platelet–large cell ratios; NPV, negative predictive value; PPV, positive predictive value; ROC, receiver operating characteristic.

## Discussion

Preeclampsia, a condition unique to pregnancy affecting multiple organs, ranks as the second leading cause of maternal mortality globally.^14^ Research indicates that three out of five maternal deaths in the United States are linked to the misidentification or delayed diagnosis of this condition.^15^ Consequently, early prediction of preeclampsia is vital for the well-being of both mothers and fetuses. Given the varying levels of health care access across regions and the increasing incidence of preeclampsia, discovering cost-effective and readily accessible predictors for preeclampsia is of immense importance.^6^ In fact, studies have found that mean arterial pressure obtained as early as the 13th week of pregnancy is an effective predictor of preeclampsia in women with normal blood pressure before pregnancy, but urinary protein is not.^16^ However, blood pressure monitoring is susceptible to environmental, emotional, and genetic factors, and it is very passive to predict preeclampsia as a symptom. Platelet activation is evidently intertwined with the development of preeclampsia,^17^ and this activation may manifest weeks before the onset of the condition.^18^ Hence, we conducted this retrospective study, meticulously reviewing the obstetric medical histories of participants, to investigate the predictive potential of platelet parameters both before and after the 16th week of pregnancy for preeclampsia.

Vascular inflammation and endothelial dysfunction, pivotal factors in the onset of preeclampsia, lead to abnormal activation of the fibrinolytic system and platelets. This, in turn, results in heightened peripheral platelet consumption and perturbed regulation of bone marrow hematopoiesis, precipitating alterations in platelet parameters.^9^ This could explain the observed elevation in platelet parameters in the serum of pregnant women with preeclampsia during the second trimester of pregnancy in our study.

Several studies have noted a significant decrease in PC in preeclampsia groups compared with control groups, potentially stemming from vascular endothelial damage in preeclampsia. This damage may lead to increased platelet depletion and turnover, prompting the establishment of PC as a predictive marker for preeclampsia.^11,13,19–21^ However, conflicting studies have contradicted these findings, suggesting that reduced PC may be a consequence of pregnancy itself rather than preeclampsia.^22^ Such discrepancies in conclusions may arise from variations in sample sizes or the gestational weeks at which samples were collected.

Reports indicate that an elevated MPV in the late first trimester of pregnancy can serve as an indicator for predicting both preeclampsia and intrauterine growth restriction.^23^ However, the predictive ability of MPV and PC for preeclampsia yields contrasting conclusions. Some studies affirm that MPV significantly increases in the preeclampsia group, attributing this phenomenon to the release of larger and younger platelets from the bone marrow to compensate for peripheral platelet consumption.^22,24–26^ Conversely, certain studies fail to discern this distinction.^27–29^ The disparities likely arise from variations in sample collection times and the use of EDTA anticoagulation tubes.^13^ Research has shown that EDTA can induce changes in platelet cell membranes, shifting platelet morphology from the usual disc-like shape to a spherical form, altering the conformation of specific hidden antigens on platelet surfaces.^30^ Therefore, although PC and MPV are valuable observational markers for preeclampsia, they cannot serve as definitive indicators.^31^

In our investigation, we observed no significant alterations in PC between the preeclampsia groups and the non-preeclampsia group. However, MPV, P-LCR, and PDW exhibited progressive increases with the severity of preeclampsia in the preeclampsia groups. Based on our univariate analysis and logistic regression findings, we conclude that P-LCR and PDW possess independent predictive potential for preeclampsia. This aligns with prior research indicating that endothelial damage-induced platelet activation leads to an increase in immature platelets and platelet turnover, culminating in elevated P-LCR and PDW in preeclamptic pregnant women.^11,19,32^ Furthermore, placental hypoxia in pregnant women with preeclampsia can stimulate the release of erythropoietin by the kidney and liver, triggering the production of large megakaryocytes in the bone marrow, thereby influencing platelet parameters.^20^

It is important to acknowledge that our study, being retrospective, is subject to certain selection biases. In addition, our findings are derived from a central study and may lack generalizability. At the same time, as a cross-sectional study, the role of platelet parameters in predicting and assessing the severity of preeclampsia throughout pregnancy could not be determined. Further longitudinal studies to evaluate platelet parameters at different gestational weeks are warranted to advance the identification of effective predictors for preeclampsia. In summary, P-LCR and PDW hold potential predictive value for preeclampsia in clinical practice.

## Ethical Statement

All procedures involving human participants in this study comply with the Declaration of Helsinki (revised in 2013). This study was approved by the Logic Committee of the Affiliated Hospital of Jining Medical College and obtained informed consent from all participants.

## Consent to Publish

Human participants have provided informed consent for the images and tables published in the article.

## Abbreviations Used

BMI: body mass index
CI: confidence interval
EDTA: ethylene diamine tetraacetic acid
MPV: mean platelet volume
NPV: negative predictive value
OR: odds ratio
P: probability value
PC: platelet count
PDW: platelet distribution width
PE: preeclampsia
P-LCR: platelet large cell ratios
PPV: positive predictive value
ROC: receiver operating characteristic
